# Trends in the US and Canadian Pathologist Workforces From 2007 to 2017

**DOI:** 10.1001/jamanetworkopen.2019.4337

**Published:** 2019-05-31

**Authors:** David M. Metter, Terence J. Colgan, Stanley T. Leung, Charles F. Timmons, Jason Y. Park

**Affiliations:** 1 Department of Pathology, University of Texas Southwestern Medical Center, Dallas; 2 Department of Pathology, Children’s Health, Dallas, Texas; 3 Department of Lab Medicine & Pathobiology, University of Toronto, Toronto, Ontario, Canada; 4 LifeLabs, Toronto, Ontario, Canada; 5 Incyte Diagnostics, Seattle, Washington; 6 Eugene McDermott Center for Human Growth and Development, University of Texas Southwestern Medical Center, Dallas

## Abstract

**Importance:**

The current state of the US pathologist workforce is uncertain, with deficits forecast over the next 2 decades.

**Objective:**

To examine the trends in the US pathology workforce from 2007 to 2017.

**Design, Setting, and Participants:**

A cross-sectional study was conducted comparing the number of US and Canadian physicians from 2007 to 2017 with a focus on pathologists, radiologists, and anesthesiologists. For the United States, the number of physicians was examined at the state population level with a focus on pathologists. New cancer diagnoses per pathologist were compared between the United States and Canada. These data from the American Association of Medical Colleges Center for Workforce Studies’ Physician Specialty Data Books and the Canadian Medical Association Masterfile were analyzed from January 4, 2019, through March 26, 2019.

**Main Outcomes and Measures:**

Numbers of pathologists were compared with overall physician numbers as well as numbers of radiologists and anesthesiologists in the United States and Canada.

**Results:**

Between 2007 and 2017, the number of active pathologists in the United States decreased from 15 568 to 12 839 (−17.53%). In contrast, Canadian data showed an increase from 1467 to 1767 pathologists during the same period (+20.45%). When adjusted for each country’s population, the number of pathologists per 100 000 population showed a decline from 5.16 to 3.94 in the United States and an increase from 4.46 to 4.81 in Canada. As a percentage of total US physicians, pathologists have decreased from 2.03% in 2007 to 1.43% in 2017. The distribution of US pathologists varied widely by state; per 100 000 population, Idaho had the fewest (1.37) and the District of Columbia had the most (15.71). When adjusted by new cancer cases per year, the diagnostic workload per US pathologist has risen by 41.73%; during the same period, the Canadian diagnostic workload increased by 7.06%.

**Conclusions and Relevance:**

The US pathologist workforce decreased in both absolute and population-adjusted numbers from 2007 to 2017. The current trends suggest a shortage of US pathologists.

## Key Points

**Question:**

What is the current state of the US pathologist workforce compared with the Canadian pathologist workforce?

**Findings:**

In this cross-sectional study, a 17.53% decrease in the number of US pathologists was identified from 2007 to 2017. During the same period, there was a 20.45% increase in the number of Canadian pathologists.

**Meaning:**

Adjusted for population, the US pathologist workforce is now smaller relative to other countries that have experienced major adverse events in clinical laboratory quality and delays in diagnosis.

## Teaser

This cross-sectional study examines changes in the number of pathologists compared with other physicians practicing in the United States and Canada from 2007 to 2017.

## Introduction

Pathologist shortages in the Canadian and UK health systems have resulted in suboptimal patient care, including delayed cancer diagnoses and diagnostic errors.^1,2,3^ A 2017 survey conducted by the UK Royal College of Pathologists found adequate staffing in only 3% of National Health Service histopathology departments.^1^ This inadequate staffing has resulted not only in diagnostic delays but also in increased costs due to the need to hire temporary workers or outsource services. Because of the potential consequences of a pathologist shortage, a comprehensive understanding of the current and future pathologist workforce is imperative. In contrast to other medical specialties, in which the public can directly observe inadequate staffing via delays in physician scheduling, the pathologist workforce is unique in that workload imbalance is not directly observed by patients and the public until a critical event occurs such as a diagnostic error or long delays in diagnosis. Indeed, clinical specialties have a maximum workload responsibility limited by the number of patients they can see and treat.^4^ In contrast, in many laboratories, pathologists are responsible for all clinical specimens and materials they receive from clinical colleagues in a given period of time. Thus, a pathologist’s workload is not capped by a specific number of patients, but rather expands to encompass all case materials generated by their clinical colleagues. The dangers of overwork, diminishing quality, and diagnostic error are all reported in pathology.^5^ In this study, we broadly examine the trends of the total pathologist workforce in the United States and use the Canadian pathologist workforce as a reference.

## Methods

This study was evaluated by the institutional review board of the University of Texas Southwestern Medical Center and was not considered human subjects research. This study followed the Strengthening the Reporting of Observational Studies in Epidemiology (STROBE) reporting guideline for cross-sectional studies.^6^

### US Pathologist Data

The Association of American Medical Colleges (AAMC) Physician Specialty Data Books (2008-2018) were the primary data source.^7,8,9,10,11,12^ The AAMC Data Book is a biennial publication that contains the previous year’s data; for example, the 2008 Physician Specialty Data Book contains 2007 statistics. The AAMC report is based on the American Medical Association (AMA) Masterfile, which includes current and historical data for more than 1 million physicians, residents, and medical students in the United States. Included in the data are both allopathic and osteopathic physicians as well as approximately 500 000 US practicing physicians who graduated from medical schools outside the United States (international medical graduates). An individual’s record begins when he or she enters medical school (US allopathic graduate) or an Accreditation Council for Graduate Medical Education–accredited residency training program (allopathic, osteopathic, or international graduate). The Masterfile also includes information on US state licensure, National Provider Identification registry, and professional board certification. Importantly, individual Masterfile records are specifically tracked for deceased physicians to prevent fraud or identity theft. Relevant to this study, the Masterfile records a physician’s self-designated practice specialty. In this study, *pathology* refers to the AAMC category of anatomic/clinical pathology, which includes anatomic pathology, anatomic/clinical pathology, chemical pathology, and clinical pathology. Active physician data for radiology and anesthesiology are used for reference. Beginning in 2013 (2014 Data Book), radiology encompassed 3 tracked specialties: diagnostic radiology, neuroradiology, and vascular/interventional radiology; for this study, these 3 categories are combined into 1 group, radiology. The AAMC also provides physician data from each state. In this study, the AAMC State Physician Workforce Data Books for 2013 and 2017 are used; these Data Books correspond to physician data from the years 2012 and 2016, respectively. Trends in number of US pathologists from 2007 to 2017 are compared with overall physician numbers, other specialties (radiology and anesthesiology), population (state and national), and new cancer diagnoses.

### Canadian Pathologist Data

The Canadian Medical Association (CMA) conducts an annual survey of active physicians across multiple specialties.^13^ For equivalence to US tracking, this study will refer to the CMA Masterfile category of laboratory medicine specialists when describing Canadian pathologists. The category of laboratory medicine specialists encompasses the physician categories of anatomical pathology, forensic pathology, general pathology (equivalent to US anatomic/clinical pathology), hematologic pathology, medical biochemistry, medical microbiology, and neuropathology. In the CMA Masterfile, radiology was tracked as 2 categories beginning in 2014: diagnostic radiology and neuroradiology. In 2017, radiology was tracked as 3 categories: diagnostic radiology, neuroradiology, and pediatric radiology. We compared US and Canadian data for differences in trends from 2007 to 2017 in pathologists and other specialties (radiology and anesthesiology). We compared US and Canadian pathologists with respect to overall population, total number of physicians, and new cancer diagnoses per pathologist.

### Population and New Cancer Diagnoses Data

Country-level population data from 2007 to 2017 were extracted from the US Census Bureau (Population Total, American Community Survey, and American Fact Finder)^14^ and Statistics Canada.^15^ Annual new cancer diagnosis estimates were taken from the American Cancer Society^16,17,18,19,20^ and the Canadian Cancer Society.^21^

### Statistical Analysis

Data were analyzed from January 4, 2019, through March 26, 2019. Absolute numbers of individual physicians or cancer cases were used. For population comparison, the number of physicians or cancer cases was adjusted for national or state populations. Excel 2016 (Microsoft Corp) was used for calculations (range, percentage, and per capita calculations) and graphing.

## Results

**Figure 1.**
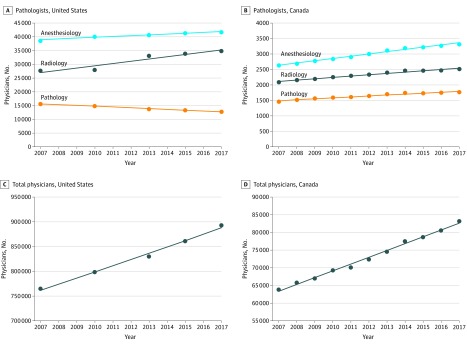
Decline in Number of US Pathologists from 2007 to 2017 A, The total number of pathologists in the United States decreased in each year from 2007 to 2017, for an overall decrease of 17.53% (from 15 568 to 12 839). In contrast, numbers of US anesthesiologists and radiologists showed overall growth in the same 10-year period. B, In Canada, the total number of pathologists grew 20.45% (from 1467 to 1767), which was comparable to growth observed in numbers of Canadian anesthesiologists and radiologists. C, The total number of US physicians increased by 16.61% (from 765 688 to 892 856). D, The total number of Canadian physicians in this period increased by 30.30% (from 63 819 to 83 159).

**Figure 2.**
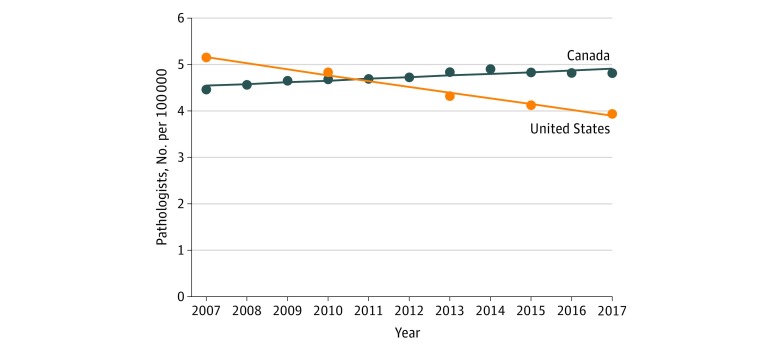
Pathologists per 100 000 Population, United States vs Canada The numbers of pathologists in the United States and Canada were adjusted per 100 000 population of the respective countries from 2007 to 2017. In 2007, there were 5.16 and 4.46 pathologists per 100 000 population in the United States and Canada, respectively. In 2017, there were 3.94 and 4.81 pathologists per 100 000 population in the United States and Canada, respectively.

During the period of 2007 to 2017, the US physician workforce grew from 765 688 to 892 856 (+16.61%); however, the pathologist workforce decreased from 15 568 to 12 839 (−17.53%) (Figure 1A; eTable 1 in the Supplement). This loss of 2729 US pathologists occurred with a decrease ranging between −3.09 and −8.45% with each report period. Anesthesiologists and radiologists in the United States both experienced overall growth of 7.85% and 26.32%, respectively, over the same 10-year period. As a percentage of total US physicians, pathologists decreased from 2.03% to 1.43%. Over the same period, the Canadian pathologist workforce increased from 1467 to 1767 (+20.45%) within a total Canadian physician workforce growth from 63 819 to 83 159 (+30.30%) (Figure 1B; eTable 2 in the Supplement). Canadian anesthesiologists and radiologists experienced growth of 26.26% and 20.40%, respectively, during this period. As a percentage of total physicians, Canadian pathologists decreased from 2.30% to 2.12% of physicians. When adjusted for population, the number of US pathologists declined from 5.16 to 3.94 pathologist per 100 000 population (−23.64%) (Figure 2; eTable 3 in the Supplement). In comparison, the number of Canadian pathologists per 100 000 population increased from 4.46 to 4.81 (+7.85%) (Figure 2; eTable 4 in the Supplement).

**Figure 3.**
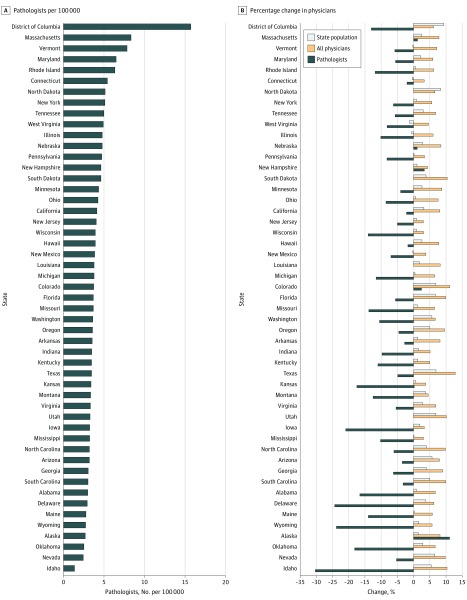
Numbers of Pathologists per 100 000 Population and Percentage Change by State A, The number of pathologists per state (including the District of Columbia) in 2016, adjusted for 100 000 of population, shows variation from 15.71 for the District of Columbia to 1.37 for Idaho. B, The change in pathologists from 2012 to 2016 was mostly negative, with only 9 states showing zero or positive growth. In contrast, all states (including the District of Columbia) showed growth in overall physician numbers (range, 3.02%-12.86%). Most state populations grew (range, 0.13%-9.28%), with only 5 states showing declining populations.

The trend in US pathologist workforce was further examined at the state level for the years 2012 and 2016 (Figure 3; eTable 5 in the Supplement).^22,23^ In 2016, the number of pathologists per 100 000 state population varied widely, with Idaho having the fewest (1.37) and the District of Columbia having the most (15.71); the state median was 3.68 pathologists per 100 000 population. When change in number of pathologists was compared between 2012 and 2016, most states showed negative growth, ranging from −1.75% in Hawaii to −30.30% in Idaho. Four states showed zero growth: Louisiana, North Dakota, South Dakota, and Utah. Five states showed positive growth, ranging from +1.11% in Nebraska to +11.11% in Alaska. Interestingly, during this same period, every state had positive physician growth, ranging from +3.02% to +12.86%. Most states had positive overall population growth, ranging from +0.13% to +9.28%. Only 5 states had negative population growth: Connecticut, Illinois, New Mexico, Vermont, and West Virginia. The 3 states with the largest percentage decline in pathologists (Idaho, Delaware, and Wyoming) had contrasting positive growth in both population and overall number of physicians.

**Figure 4.**
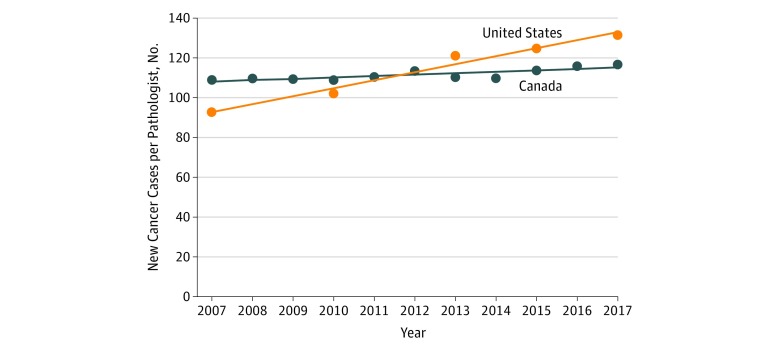
Increasing New Cancer Cases per Pathologist, United States vs Canada The numbers of pathologists in the United States and Canada were adjusted for the new cancer diagnoses of the respective countries from 2007 to 2017. In 2007, there were 92.81 and 109.00 new cancer cases per pathologist in the United States and Canada, respectively. In 2017, there were 131.54 and 116.69 new cancer cases per pathologist in the United States and Canada, respectively.

Finally, during the 10-year study period, the estimated number of new cancer cases in the United States increased from 1 444 920 to 1 688 780 (+16.88%) (eTable 6 in the Supplement). When adjusted per number of US pathologists in each year, the number of cases per pathologist increased from 92.81 to 131.54 (+41.73%) (Figure 4). In comparison, over the same period, the number of new cancer cases in Canada increased from 159 900 to 206 200 (+28.96%). When adjusted per number of Canadian pathologists in each year, the number of cases per pathologist increased from 109.00 to 116.69 (+7.06%).

## Discussion

In 1 decade, the number of pathologists in the United States has decreased by 17.53% (Figure 1). This decrease is the largest of all physician specialties tracked by the AAMC workforce survey. Furthermore, when adjusted for population, the US pathologist workforce was previously greater than Canada’s workforce from 2007 to 2010 (Figure 2). However, a steady decline in the US pathology workforce combined with a modest increase in the Canadian pathology workforce has led to a smaller US workforce per population compared with the Canadian workforce. The gap between the US and Canadian workforces continues to grow. In 2017 there were only 3.94 US pathologists per 100 000 population compared with Canada’s 4.81 per 100 000 population. When examining state-specific US data (2016), the number of pathologists varied widely per 100 000 population, from 1.37 (Idaho) to 15.71 (District of Columbia) (Figure 3). The decrease in number of US pathologists is not only anomalous when compared with Canada, but also contrasts with increases in other hospital-based specialties (anesthesiology and radiology, for example) as well as the overall number of physicians.

An examination of Canadian workforce studies may provide insight into the diverging workforce trends in the United States and Canada. A study^24^ of the Canadian pathologist workforce from 1998 to 2008 showed a decreasing supply of pathologists with respect to population. Data from this current 2007 to 2017 study indicate that the previously decreasing supply of Canadian pathologists per population has been corrected and reversed; however, the cause of the increasing supply of pathologists in Canada compared with the United States in the last decade is uncertain. The Canadian pathology practice environment has evolved considerably over the last 20 years and differs significantly from that of the United States. These differences in Canadian pathology practice include a single-payer system, advanced regionalization of pathology laboratories with associated subspecialty development and academic affiliation, provincewide screening and cancer programs, and a predominance of salary and contractual arrangements. In addition, multiple adverse pathology events and trends previously received widespread attention in the Canadian media and courts.^25,26,27,28^ These adverse events combined with a highly structured and regulated laboratory system may have led to increased awareness of the key role of pathologists in the health care and legal systems.

In the present study, 2 similar trends were identified for both American and Canadian pathologists. First, American and Canadian pathologists face increased numbers of cancer cases compared with historical comparators (Figure 4). Second, as a relative proportion of total physicians, both US and Canadian pathologists decreased in number from 2007 to 2017. These trends had been identified in earlier studies^24,29^ of the Canadian pathologist workforce during 1998 to 2008.

Prior pathologist workforce studies have not used the AMA Masterfile (the database used to generate AAMC Physician Specialty Data Books). The limitations of the AMA Masterfile include difficulty in determining when a physician has retired, low response rates to AMA surveys, and delays in accounting for deceased physicians. For US pathologists, alternative data sets have been developed and published.^30^ Regardless of its potential deficiencies, the AMA Masterfile is extensively used by government agencies, insurers, and physician credentialing services.^10,31,32^ A key strength of the AMA Masterfile is that it has been regularly updated and provides information relative to other medical specialties in the United States. Indeed, the most frequent criticism of the AMA Masterfile is that it overestimates the number physicians tracked.^33^

A 2013 pathologist workforce study^30^ that used an alternative data set to the AMA Masterfile estimated that there were 17 986 pathologists (17 570 full time equivalent [FTE]) in 2010 and predicted that the number of FTE pathologists would decline to 14 063 by 2030; the present study using AAMC physician data based on the AMA Masterfile estimated 14 975 pathologists (individuals, not FTE) in 2010. Addressing this 21% discrepancy between the 2 data sets should be the subject of future studies. Unfortunately, the alternative data set is not publicly available and has not been updated since 2013. A follow-up study^34^ to the 2013 pathology workforce analysis predicted a supply shortage of pathologists due to an aging US population and anticipated retirements from the pathology workforce. Based on a computational model, they forecasted a deficit of 5000 pathologist FTEs by the year 2030.

While the decreasing number of pathologists would be expected to cause widespread workforce deficits or position vacancies, a recent job market survey from the College of American Pathologists Graduate Medical Education Committee^35^ found that new trainees had difficulty finding jobs, but otherwise the pathologist job market was stable. Furthermore, an examination of 5 years of pathology job advertisements (2013-2017) also demonstrated a stable US pathology job market.^36^ In physician salary surveys from 2011 through 2017, the average salary of pathologists had an overall increase of 29%, compared with 27% and 25% reported by radiologists and anesthesiologists, respectively (eTable 7 in the Supplement).^37,38,39,40,41,42,43^

Our study does not resolve the discrepancy between a shrinking workforce without corresponding marked increase in open positions or salaries. There are likely several factors contributing to a disconnect between a diminishing supply of pathologists without a matched increase in marketplace demand for new pathologists. One such factor is increased efficiency spurred by advancements in information technology (eg, laboratory information systems, electronic health records), management practices (eg, multihospital health systems), and practice models (eg, regional reference laboratories serving multiple hospitals). A single pathologist can now direct more laboratories and can interpret more cases than was previously possible. With these advancements, a smaller workforce is taking on an increasing workload. We hypothesize that the 17.53% decrease in active pathologists has been absorbed by increased efficiency, or at least increased elasticity in pathologist work tolerance. With new technologies and practice models, a smaller pathologist workforce may be able to perform the same amount of work as their predecessors. Currently, these advances in efficiency are likely to continue through surgical pathology subspecialization, expanded use of nonphysician extenders (ie, pathology assistants and histotechnologists), and digital imaging of slides to facilitate slide review at distant or remote sites without the need for travel. As for the future of the pathologist workforce, there are new technologies on the horizon that may further decrease the need for pathologists. These emerging technologies include artificial intelligence (eg, diagnosis aide, report writing) and new health care delivery models (eg, direct to consumer, wearable diagnostic devices, and improved imaging technologies). Consequently, the development of an international benchmark for the ideal pathologist to population ratio is challenging, if not impossible. Multiple factors (eg, scope of practice, supportive infrastructure) determine work demands and efficiencies and, ultimately, the number of pathologists needed. The capacity for pathologist overwork in the United States is not well understood, and it is possible that US pathologists can continue to absorb further reductions in the workforce.

Although improved efficiency may be an explanation for an absorption of an increasing workload, caution needs to be observed to avoid potential overwork and burnout. The potential for pathologist overwork is acute because pathologists cannot determine the number of patients serviced. A recent Canadian study^4^ showed that patient safety is affected when pathologists work more than 39 hours per week. Pathologist fatigue and burnout are key contributors to errors in pathology.^5^ A Swiss study^44^ identified pathologist occupational health issues including musculoskeletal problems (40%) and workplace injuries (83%). Poor pathologist health and well-being may contribute to patient safety issues, including increased turnaround time of diagnoses and decreased quality of patient care.

Finally, a potential solution to a physician workforce shortage is to increase the number of residency training programs and positions. In our opinion, this should be approached with caution for several reasons. First, the necessary increase would be too large to be supported by existing residency programs. The number of new board-certified pathologists entering the workforce is approximately 600 per year.^7,8,9,11,12^ However, the current data reveal an average net loss of approximately 270 pathologists per year beyond that number. Thus, to simply offset the current rate of attrition, residency positions would need to increase by 45% (600 existing resident slots per year plus an additional 270). Second, from 2007 to 2017, there has been a decline in first-year residents in US pathology programs from 609 to 599 (−2%) (eTable 8 in the Supplement).^7,8,9,11,12^ In comparison, the number of first-year residents in radiology and anesthesiology has increased by 42% and 10%, respectively. Along with the slight decrease in first-year pathology residents, the percentage of residents and fellows training in pathology with international medical degrees has increased from 29.60% to 43.73%. For both radiology and anesthesiology (residents and fellows), the percentage of international medical graduates has been consistently less than 16%. Unlike radiology and anesthesiology, pathology training programs rely heavily on international medical graduates. It is unclear whether international medical graduates could sustain the theoretical 45% increase in pathology residency trainees needed to offset the current decline in active pathologists. A better understanding of the pathology specialty is needed with regard to medical student residency selection, career opportunities, and retirement.

### Limitations

This study has limitations. The US and Canadian data for this cross-sectional study each rely on single data sets: the AAMC Physician Specialty Data Books (AMA Masterfile) and the CMA Masterfile, respectively. Additional studies should validate these findings by identifying and studying alternative data sets of physician demographics. In addition, because this was a cross-sectional study, the causes and downstream effects of the decreasing workforce are not identified. Although data from other countries indicate that the current number of US pathologists may be insufficient, the current study does not examine the possibility that a decreased US pathologist workforce may provide efficient service at equivalent or higher quality compared with historical or international norms.

## Conclusions

The US pathologist workforce is now, per capita, smaller than the Canadian pathologist workforce. Given the potential negative health care consequences of a pathologist shortage, efforts to understand the etiology of the shrinking US pathologist workforce should be initiated by policy makers, health care delivery systems, insurers, and physicians. Because the Canadian pathologist workforce has increased during this period of a shrinking US workforce, lessons from the Canadian experience may provide valuable insights into further action in the United States.

## Notes

Supplement. **eTable 1.** US Active Physicians **eTable 2.** Canadian Active Physicians **eTable 3.** US Pathologists per 100,000 **eTable 4.** Canadian Pathologists per 100,000 **eTable 5.** US Pathologists by State in 2012 and 2016 **eTable 6.** Cancer Cases per 100,000 for US and Canada **eTable 7.** Surveyed Average Salaries (US Dollars) **eTable 8.** PGY1 Residents and Percentage International Medical Degree Residents/Fellows
