# Supplementary material for: Trends in the US and Canadian Pathologist Workforces From 2007 to 2017
Source: JAMA Netw Open. 2019 May 31;2(5):e194337. doi: 10.1001/jamanetworkopen.2019.4337 (PMC6547243; doi:10.1001/jamanetworkopen.2019.4337)
Supplement: Supplement. — eTable 1. US Active Physicians eTable 2. Canadian Active Physicians eTable 3. US Pathologists per 100,000 eTable 4. Canadian Pathologists per 100,000 eTable 5. US Pathologists by State in 2012 and 2016 eTable 6. Cancer Cases per 100,000 for US and Canada eTable 7. Surveyed Average Salaries (US Dollars) eTable 8. PGY1 Residents and Percentage International Medical Degree Residents/Fellows [file jamanetwopen-2-e194337-s001.pdf]

Supplementary Online Content

Metter DM, Colgan TJ, Leung ST, Timmons CF, Park JY. Trends in the US and Canadian pathologist workforces from 2007 to 2017. *JAMA Netw Open*. 2019;2(5):e194337. doi:10.1001/jamanetworkopen.2019.4337

eTable 1. US Active Physicians

eTable 2. Canadian Active Physicians

eTable 3. US Pathologists per 100,000

eTable 4. Canadian Pathologists per 100,000

eTable 5. US Pathologists by State in 2012 and 2016

eTable 6. Cancer Cases per 100,000 for US and Canada

eTable 7. Surveyed Average Salaries (US Dollars)

eTable 8. PGY1 Residents and Percentage International Medical Degree Residents/Fellows

This supplementary material has been provided by the authors to give readers additional information about their work.

© 2019 Metter DM et al. *JAMA Network Open.*

eTable 1: US Active Physicians

	Pathology	Radiology	Anesthesiology	All Active Physicians	Pathology (%)
**2007**	15,568	27,562	38,724	765,688	2.03
**2008**					
**2009**					
**2010**	14,975	27,986	40,123	799,502	1.87
**2011**					
**2012**					
**2013**	13,710	33,068	40,758	829,962	1.65
**2014**					
**2015**	13,286	33,784	41,351	860,939	1.54
**2016**					
**2017**	12,839	34,817	41,762	892,856	1.43
**Absolute Difference 2007 to 2017**	-2,729	7,255	3,038	127,168	
**Percent Difference 2007 to 2017**	**-17.53**	26.32	7.85	**16.61**	
	**Pathologist (% change)**			**All Active Physicians (% change)**	
**2007-10**	-3.81			+4.42	
**2010-13**	-8.45			+3.81	
**2013-15**	-3.09			+3.73	
**2015-17**	-3.36			+3.71	
**Average**	-4.68			+3.92	

Note: AAMC Physician Specialty Data Books (2008, 2012, 2014, 2016, 2018). Beginning in the 2014 Data Report (2013 data), Radiology tracked with 3 categories: Diagnostic Radiology, Neuroradiology, and Vascular & IR.

© 2019 Metter DM et al. *JAMA Network Open.*

eTable 2: Canadian Active Physicians

	Pathology	Radiology	Anesthesiology	All Active Physicians	Pathology (%)
**2007**	1467	2087	2628	63819	2.30
**2008**	1517	2153	2698	65794	2.31
**2009**	1563	2193	2782	66992	2.33
**2010**	1591	2248	2843	69267	2.30
**2011**	1609	2294	2914	70088	2.30
**2012**	1643	2334	3009	72368	2.27
**2013**	1700	2396	3118	74526	2.28
**2014**	1742	2463	3201	77479	2.25
**2015**	1731	2466	3230	78657	2.20
**2016**	1747	2477	3274	80544	2.17
**2017**	1767	2534	3318	83159	2.12

Note: CMA Masterfile (2007-2017). Beginning in 2014, Radiology tracked with 2 categories: Diagnostic Radiology and Neuroradiology. Beginning in 2017, Radiology tracked with 3 categories: Diagnostic Radiology, Neuroradiology and Pediatric Radiology

© 2019 Metter DM et al. *JAMA Network Open.*

eTable 3: US Pathologists per 100,000

	US Pathologists	US population	Pathologist per 100000	pathologist per capita
**2007**	15,568	301621159	5.16	1:19374
**2008**		304059728		
**2009**		307006556		
**2010**	14,975	309349689	4.84	1:20658
**2011**		311591919		
**2012**		313914040		
**2013**	13,710	316128839	4.34	1:23058
**2014**		318857056		
**2015**	13,286	321418821	4.13	1:24192
**2016**		323127515		
**2017**	12,839	325719178	3.94	1:25370

© 2019 Metter DM et al. *JAMA Network Open.*

eTable 4: Canadian Pathologists per 100,000

	Canada pathologists	Canada population	Pathologist per 100000	pathologist per capita
**2007**	1,467	32887928	4.46	22418
**2008**	1,517	33245773	4.56	21915
**2009**	1,563	33628571	4.65	21515
**2010**	1,591	34005274	4.68	21374
**2011**	1,609	34342780	4.69	21344
**2012**	1,643	34750545	4.73	21151
**2013**	1,700	35152370	4.84	20678
**2014**	1,742	35535348	4.90	20399
**2015**	1,731	35832513	4.83	20700
**2016**	1,747	36264604	4.82	20758
**2017**	1,767	36708083	4.81	20774

© 2019 Metter DM et al. *JAMA Network Open.*

eTable 5: US Pathologists by State in 2012 and 2016

	2012			2016				2016-2012		
State	Phys	State Pop	Path	Phys	State Pop	Path	Path per 100000	Path diff	Phys diff	State pop diff
Alabama	9681	4822023	175	10329	4863300	146	3.00	-16.57%	6.69%	0.86%
Alaska	1813	731449	18	1961	741894	20	2.70	11.11%	8.16%	1.43%
Arizona	15133	6553255	230	16345	6931071	222	3.20	-3.48%	8.01%	5.77%
Arkansas	5629	2949131	109	6088	2988248	106	3.55	-2.75%	8.15%	1.33%
California	97977	38041430	1655	105907	39250017	1619	4.12	-2.18%	8.09%	3.18%
Colorado	13869	5187582	202	15422	5540545	207	3.74	2.48%	11.20%	6.80%
Connecticut	11949	3590347	198	12341	3576452	194	5.42	-2.02%	3.28%	-0.39%
D.C.	5559	623323	123	5901	681170	107	15.71	-13.01%	6.15%	9.28%
Delaware	2439	917092	37	2592	952065	28	2.94	-24.32%	6.27%	3.81%
Florida	48852	19317568	803	53685	20612439	758	3.68	-5.60%	9.89%	6.70%
Georgia	21300	9919945	336	23215	10310371	315	3.06	-6.25%	8.99%	3.94%
Hawaii	4037	1392313	57	4350	1428557	56	3.92	-1.75%	7.75%	2.60%
Idaho	2938	1595728	33	3241	1683140	23	1.37	-30.30%	10.31%	5.48%
Illinois	33874	12875255	682	35927	12801539	613	4.79	-10.12%	6.06%	-0.57%
Indiana	14295	6537334	257	15025	6633053	232	3.50	-9.73%	5.11%	1.46%
Iowa	6414	3074186	129	6627	3134693	102	3.25	-20.93%	3.32%	1.97%
Kansas	6151	2885905	120	6380	2907289	99	3.41	-17.50%	3.72%	0.74%
Kentucky	9678	4380415	173	10158	4436974	154	3.47	-10.98%	4.96%	1.29%
Louisiana	10846	4601893	177	11737	4681666	177	3.78	0.00%	8.22%	1.73%
Maine	4084	1329192	43	4320	1331479	37	2.78	-13.95%	5.78%	0.17%
Maryland	21455	5884563	414	22731	6016447	391	6.50	-5.56%	5.95%	2.24%
Massachusetts	28016	6646144	561	30213	6811779	568	8.34	1.25%	7.84%	2.49%
Michigan	26476	9883360	424	28206	9928300	375	3.78	-11.56%	6.53%	0.45%
Mississippi	5396	2984926	108	5562	2988726	97	3.25	-10.19%	3.08%	0.13%
Missouri	15282	6021988	260	16268	6093000	224	3.68	-13.85%	6.45%	1.18%
Minnesota	14814	5379139	250	16105	5519952	240	4.35	-4.00%	8.71%	2.62%
Montana	2297	1005141	40	2401	1042520	35	3.36	-12.50%	4.53%	3.72%
Nebraska	4080	1855525	90	4426	1907116	91	4.77	1.11%	8.48%	2.78%
Nevada	5361	2758931	76	5884	2940058	72	2.45	-5.26%	9.76%	6.57%
New Hampshire	3942	1320718	60	4113	1334795	62	4.64	3.33%	4.34%	1.07%
New Jersey	25604	8864590	381	26378	8944469	362	4.05	-4.99%	3.02%	0.90%
New Mexico	4839	2085538	86	5023	2081015	80	3.84	-6.98%	3.80%	-0.22%
New York	68273	19570261	1078	72095	19745289	1011	5.12	-6.22%	5.60%	0.89%
North Carolina	23034	9752073	347	25295	10146788	326	3.21	-6.05%	9.82%	4.05%
North Dakota	1651	699628	39	1759	757952	39	5.15	0.00%	6.54%	8.34%
Ohio	31248	11544225	541	33621	11614373	495	4.26	-8.50%	7.59%	0.61%
Oklahoma	7552	3814820	121	8057	3923561	99	2.52	-18.18%	6.69%	2.85%
Oregon	10995	3899353	154	12050	4093465	147	3.59	-4.55%	9.60%	4.98%
Pennsylvania	38565	12763536	658	39863	12784227	604	4.72	-8.21%	3.37%	0.16%
Rhode Island	3548	1050292	76	3770	1056426	67	6.34	-11.84%	6.26%	0.58%
South Carolina	10250	4723723	155	11269	4961119	150	3.02	-3.23%	9.94%	5.03%
South Dakota	1846	833354	40	2038	865454	40	4.62	0.00%	10.40%	3.85%
Tennessee	15568	6456243	352	16627	6651194	332	4.99	-5.68%	6.80%	3.02%
Texas	54167	26059203	1009	61132	27862596	960	3.45	-4.86%	12.86%	6.92%
Utah	5801	2855287	100	6389	3051217	100	3.28	0.00%	10.14%	6.86%
Virginia	20647	8185687	296	22072	8411808	280	3.33	-5.41%	6.90%	2.76%
Vermont	2084	626011	52	2233	624594	49	7.85	-5.77%	7.15%	-0.23%
Washington	18395	6897012	295	19623	7288000	264	3.62	-10.51%	6.68%	5.67%
West Virginia	4466	1855413	98	4671	1831102	90	4.92	-8.16%	4.59%	-1.31%
Wisconsin	14578	5726398	264	15026	5778708	227	3.93	-14.02%	3.07%	0.91%
Wyoming	1102	576412	21	1165	585501	16	2.73	-23.81%	5.72%	1.58%

Phys = All active physicians; Pop = population; Path = pathologists

© 2019 Metter DM et al. *JAMA Network Open.*

eTable 6: Cancer Cases per 100,000 for US and Canada

	US			Canada		
Year	Pathologists	New Cancer Cases	Cancer Cases per Pathologist	Pathologists	New Cancer Cases	Cancer Cases per Pathologist
**2007**	15,568	1,444,920	92.81	1,467	159,900	109.00
**2008**				1,517	166,400	109.69
**2009**				1,563	171,000	109.40
**2010**	14,975	1,529,560	102.14	1,591	173,800	109.24
**2011**				1,609	177,800	110.50
**2012**				1,643	186,400	113.45
**2013**	13,710	1,660,290	121.10	1,700	187,600	110.35
**2014**				1,742	191,300	109.82
**2015**	13,286	1,658,370	124.82	1,731	196,900	113.75
**2016**				1,747	202,400	115.86
**2017**	12,839	1,688,780	131.54	1,767	206,200	116.69

From 2007 to 2017 there has been a 41.72% and 7.06% increase in new cancer cases per pathologist in the US and Canada, respectively.

© 2019 Metter DM et al. *JAMA Network Open.*

eTable 7: Surveyed Average Salaries (US Dollars)

Year	Pathologists	Radiologists	Anesthesiologists
**2011**	221,000	315,000	309,000
**2012**	247,000	349,000	337,000
**2013**	239,000	340,000	338,000
**2014**	267,000	351,000	358,000
**2015**	266,000	375,000	360,000
**2016**	293,000	396,000	364,000
**2017**	286,000	401,000	386,000

Medscape Physician Compensation Reports 2012, 2013, 2014, 2015, 2016, 2017, 2018. WebMD LLC.

© 2019 Metter DM et al. *JAMA Network Open.*

eTable 8: PGY1 Residents and Percentage International Medical Degree Residents/Fellows

Year	Pathology		Radiology		Anesthesiology	
	PGY1†	Total % IMG*	PGY1	Total % IMG	PGY1	Total % IMG
**2007**	609	29.60	1,109	7.10	1,432	14.30
**2008**						
**2009**						
**2010**	617	31.40	1,111	8.00	1,621	12.50
**2011**						
**2012**						
**2013**	592	35.70	1,563	8.96	1,589	11.70
**2014**						
**2015**	587	39.38	1,611	11.22	1,481	11.52
**2016**						
**2017**	599	43.73	1,577	15.13	1,573	13.00

†Post-graduate Year 1 (1st year residents); *Percent of all residents and fellows that have an international medical degree.

Note: AAMC Physician Specialty Data Books (2008, 2012, 2014, 2016, 2018). The AAMC Data Books use the AAMC/AMA National GME Census. Beginning in the 2014 Data Report (2013 data), Radiology tracked with 3 categories: Diagnostic Radiology, Neuroradiology, and Vascular & IR.

© 2019 Metter DM et al. *JAMA Network Open.*
